# Resistance of the Archaeal Community to a Severe Disturbance in an Extreme Alkaline Saline Soil

**DOI:** 10.1007/s00284-026-04895-1

**Published:** 2026-05-13

**Authors:** Yendi E. Navarro-Noya, Alejandra Cruz-Mendoza, Stephanie E. Hereira-Pacheco, Selene Gómez-Acata, Marco Luna-Guido, Luc Dendooven

**Affiliations:** 1 Centro de Investigación y de Estudios Avanzados del Instituto Politécnico Nacional, Mexico City, México; 2 Laboratory of Soil Ecology, Cinvestav, México

## Abstract

An extreme disturbance, i.e. a 1-day chloroform fumigation, had a strong impact on the bacterial community structure in an extreme saline alkaline soil, but how Archaea respond to such an event is largely unknown. Three alkaline saline soils with electrolytic conductivity (EC) between 139 and 157 dS m^− 1^ and pH 10.0–10.3 were chloroform fumigated for one day and the recovery of the archaeal community determined after 1, 5 and 10 days, while an unfumigated soil served as control. Six archaeal phyla dominated by Candidatus Halobacterota (relative abundance 96.9%) were detected, while *Natronorubrum* dominated in the unfumigated (9.7%) and fumigated extreme alkaline saline soil (8.9%). Fumigation and time had a significant effect on the archaeal community structure. Some archaeal groups, e.g. *Halovarius* sp., were strongly affected by fumigation most accentuated on day 1 and 5. It was found that chloroform fumigation had only a limited and mostly a short time effect on the archaeal groups which demonstrated their resistance to a severe disturbance in extreme adverse conditions.

**Supplementary Information:**

The online version contains supplementary material available at 10.1007/s00284-026-04895-1.

## Introduction

Soil is prone to disturbances, naturally, i.e. erosion, wildfire or extreme weather events, or due to human activity, that can have a profound effect on microbial communities [1]. It is well documented that heavy metal or hydrocarbon contamination, fire or land use change alter the soil microbial community [2]. The effect of these disturbances on microbial groups will depend on soil characteristics, the composition and abundance of the original microbial community, the type of event that occurred and if it was long-lasting or short [3]. Most studies that investigated these disturbances have centered on Bacteria [4], while the effect of these perturbations on other microorganism, such as Fungi or Archaea, are less documented [5, 6]. Most studies on the effects of disturbances on Archaea have focused on specific groups, such as ammonia-oxidizing archaea (AOA), which play key roles in particular soil processes, such as nitrogen cycling [7].

As part of biological control strategies, soils have been fumigated to manage plant-parasitic nematodes [8], impacting microorganisms involved in essential soil processes [9]. We opted to chloroform fumigate a soil as this has a profound effect on the soil microorganisms and the fumigant can easily be removed after to disturbance to study the resistance of microorganisms to it [10]. Additionally, the chloroform fumigation technique, developed in the mid-1970s to measure microbial biomass C, N, and P in soil [11], is a well-documented method whose effects on soil microbial biomass C have been extensively studied [12]. Although chloroform fumigation kills most microorganisms, it does not eliminate all of them, as fundamental processes such as carbon and nitrogen mineralization, as well as nitrification, remain active.

Woese et al. [13] demonstrated that Archaea constitute a phylogenetically distinct lineage fundamentally reshaping our understanding of evolutionary biology and therefore the tree of life. Although it is well known that Archaea participate in important soil processes, such as the production and oxidation of methane (CH_4_) under anaerobic conditions [14, 15] and the oxidation of ammonium (NH_4_^+^) [16], our knowledge of how they respond to perturbations is limited [17]. We opted to chloroform fumigate a soil as this had a strong effect on the soil bacterial population [18] and has been used to determine the microbial biomass C and N [19].

Soil of the former lake Texcoco, located in the vicinity of Mexico City, is characterized by harsh environmental conditions, such as high pH and extreme salinity [20]. Therefore, the expectation is to find a high abundance of archaea within its microbial community [21]. This soil can thus be used to study the resilience of Archaea to a perturbation under extreme conditions. The same extreme alkaline saline soils of the former lake Texcoco were used to study bacterial resistance to change [18]. As such, the effect of an extreme disturbance, i.e. chloroform fumigation, on Archaea can be compared with the effect on Bacteria. Three extreme alkaline saline soils from the same area with electrolytic conductivity ranging from EC 139 to 157 dS/m and pH from 10.0 to 10.5 were chloroform fumigated for one day and inoculated with the same soil after removal of the chloroform. The unfumigated soils served as control. The archaeal community was determined in the fumigated and unfumigated soil by 454 pyrosequencing of the archaeal 16 S rRNA gene after 0, 1, 5 and 10 days. It was hypothesized that the archaeal community would show differences between the fumigated and unfumigated soil, but the effect of the chloroform fumigation on the archaeal community would be smaller than on the bacterial community, i.e. Archaea are more resistant to changes (resilient) than Bacteria in an extreme environment. The objectives of this study were to determine (i) how the archaeal community structure was affected by chloroform fumigation, (ii) which Archaea recolonized the fumigated soil, i.e. chloroform fumigation lyses microbial cells and the released organic material provides readily available organic material and (iii) how did the chloroform fumigation affect the archaeal community as compared to the bacterial community.

## Materials and Methods

### Soil Sampling

From the 17th century onwards, lake Texcoco was drained to avoid flooding in Mexico City [20]. It is now a large area of lacustrine bed exposed to desertification as the potential evaporation rate (1800 mm/y) is larger than the rainfall (570 mm/y). The soil of the former lake Texcoco is characterized by a large water holding capacity (WHC), high pH and EC. The extreme alkaline saline soil has a pH often > 10, salinity > 150 dS/m and the WHC > 100%. Patches of salt crusts covers most of the soil surface, although *Distichlis spicata* (L.) Greene, a halophilic grass, can be found where the salinity is less extreme. Soil was collected by augering randomly 20 times the top layer (0–15 cm) from three different 400 m^2^ areas (Sample 1: 19^o^30.800’ N, 98^o^59.419’ W; Sample 2: 19^o^30.785’ N, 98^o^59.419’ W; Sample 3: 19^o^30.797’ N, 98^o^59.438’ W). The soil collected at each area was pooled so that three soil samples (*n* = 3) were obtained and characterized. The field-based replication was maintained in the laboratory experiment to avoid pseudo-replication.

### Chemical Characterization

The electrolytic conductivity (EC) was determined by mixing distilled water with a 5 g subsample of soil in a ratio 1:5 and measuring the EC with a conductometer Mettler Toledo^®^ Model S220 (New York, USA) [22]. A soil sample was mixed with distilled water in a 1:2.5 ratio and pH was determined with a potentiometer Mettler Toledo^®^ Model S220 (New York, USA) [23]. The hydrometer method as described by Gee and Bauder [24] was used to determine the soil particle size distribution, while total C was determined with a Thermo Scientific™ FlashSmart™ Elemental Analyzer (Waltham, Massachusetts, USA) following the standard protocol provided by the manufacturer. The inorganic C in soil was determined by adding 20 mL 1 M HCl solution to 2 g air-dried soil and trap CO_2_ evolved in 20 mL 1 M NaOH. The organic C was defined as the difference between the total and inorganic C. The Kjeldahl method was used to determine total N with concentrated H_2_SO_4_, K_2_SO_4_ and HgO to digest the sample [25]. The water holding capacity (WHC) WHC was measured on soil samples water-saturated in a funnel and left to stand overnight. The weight of the water retained by the soil was considered the WHC.

### Fumigation Incubation

The experimental design is summarized in Fig. S1. Sub-samples of 25 g soil (*n* = 8) of each plot (*n* = 3) were added separately to 120 mL glass flasks. Half of the flasks (*n* = 4) of each plot were placed in a desiccator containing a beaker with distilled water. The samples were fumigated with ethanol free chloroform for one day [19]. After one day, the headspace of the desiccator was vacuum evacuated for approximately 30 min so that all chloroform was removed. The flasks with the fumigated soil samples were taken from the desiccator. A 0.1 g unfumigated soil sample was mixed into the fumigated soil samples. The flasks were placed separately in 1 L glass jars containing two 25 mL beakers: one with 20 mL 1 M NaOH to trap the emitted CO_2_ and the other with distilled water to avoid desiccation of the soil samples. The soil in the other half of the flasks remained unfumigated, i.e. the unfumigated soil samples. The unfumigated soil samples were also mixed as it has been shown that mixing a soil alters the microbial community [26]. The flasks with the unfumigated soil samples were placed separately in 1 L glass jars containing a 25 mL beaker with 20 mL 1 M NaOH and one with distilled water. All the glass jars were closed airtight and incubated in the dark at 25 °C for 10 days.

After 0, 1, 5 and 10 days, one jar with unfumigated and fumigated soil was selected at random and opened. The vessel with 1 M NaOH was removed from the flask, stoppered and stored briefly. The 1 M NaOH was titrated with 0.1 M HCl to determine the CO_2_ trapped [19]. The soil microbial biomass was calculated as the [(CO_2_ emitted from the fumigated soil - CO_2_ emitted from the unfumigated soil) × 2.22] [19].

### DNA Extraction and PCR Amplification of Archaeal 16 S rRNA Genes

After 0, 1, 5 and 10 days, the incubated fumigated or unfumigated soil (4 ⋅ 0.5 g soil) was extracted for DNA based on enzymatic, physical and chemical cell lysis [27, 28]. A 0.5 g soil sample was re-suspended in a 8 mL lysis solution I (0.15 M NaCl, 0.1 M EDTA, pH 8.0, 10 mg lysozyme/mL), mixed and incubated at 37 °C for 1 h followed by a 8 mL lysis solution II (0.1 M NaCl, 0.5 M Tris-HCl, pH 8.0, 12% SDS). The soil suspension was passed through two cycles of freezing at −40 °C for 20 min and thawing at 65 °C for 20 min followed by vortex agitation for 10 min. Tubes were centrifuged at 7,700 *g* for 10 min. An equal volume of CHCl_3_:isoamyl alcohol (24:1) was added, mixed and centrifuged at 7,700 *g* for 5 min. The supernatant was transferred to a clean tube. An equal volume of 13% PEG (polyethylene glycol [8,000 MW] dissolved in 1.6 M NaCl) was added to the supernatant, incubated on ice for 30 min and centrifuged at 12,000 *g* and 4 °C for 10 min. The supernatant was decanted, the DNA pellet washed with 5 mL 70% cold ethanol and air-dried. The DNA pellet was resuspended in 500 mL deionized H_2_O. Two volumes of ethanol were added to the supernatant, mixed and centrifuged at 13,000 g and 4 °C for 30 min. The pellet was washed with 500 mL 70% cold ethanol and air-dried. The DNA extract was stored at −20 °C until used for PCR amplification.

The archaeal 16 S rRNA genes were amplified with previously reported primers 25 F [29] and A571R [30] and containing the 454 FLX adapters. The PCR mixture (25 mL) contained 1 × reaction buffer, 10 mM of each of the four deoxynucleoside triphosphates,10 pM of each of the primers, 0.7 U Phusion hot start high fidelity DNA polymerase (FINNZYMES) and 20 ng metagenomic DNA as template. The following thermal cycling scheme was used: initial denaturation at 95 °C for 10 min, 25 cycles of denaturation at 95 °C for 45 s, annealing at 53 °C for 45 s, and extension at 72 °C for 45 s followed by a final extension period at 72 °C for 10 min [31]. The product of five reactions was pooled [32] and constituted a single library. The pyrosequencing libraries were purified with the DNA Clean & Concentrator purification kit (Zymo Research, Irvine, CA, USA). The DNA was quantified using the PicoGreen^®^ dsDNA assay (Invitrogen, Carlsbad, USA) and the NanoDrop™ 3300 Fluorospectrometer (Thermo Scientific NanoDrop). The DNA was sequenced by Macrogen Inc. (DNA Sequencing Service, Seoul, Korea) with a Roche 454 GS-FLX Titanium System pyrosequencer (Roche, Mannheim, Germany).

### Bioinformatic Analyses: Taxonomic Annotation

The bioinformatic analyses of sequences were done using the “Quantitative insight into microbial ecology” v2020.8 (QIIME2) [33]. Denoising, dereplication and chimera removal was done using the DADA2 protocol [34]. This process resulted in a set of amplicon sequence variants (ASVs) dataset. Taxonomic annotation of ASVs was done using the *classify-sklearn* algorithm and using a trained database version of the Silva database v138 [35]. The Silva database training process was done with the *fit-classifier-naive-bayes* algorithm.

### Statistical Analysis

All statistical analyses were done in R v. 4.2.2 [36] within the RStudio environment (v. 2023.09.0 + 463). Alpha diversity of the bacterial community (ASVs) was determined based on the Hill numbers at different *q* orders (at *q =* 0, 1 and 2) [37]. Diversity through Hill numbers of the amplification sequence variants (ASVs) at the order *q* = 0, *q* = 1, *q* = 2 were calculated for the fumigated and unfumigated soil after 0, 1, 5 and 10 days with hillR package (v0.5.1) [38]. A non-parametric analysis with the WRS2 package (v1.1-0) [39] was used to determine the effect of fumigation and time (0, 1, 5 and 10 days) on alpha diversity (Hill numbers at the order *q* = 0, 1 and 2). Beta-diversity of the effect of fumigation (beta.pair: fumigated and unfumigated soil) and changes over time (beta.temp: 0, 1, 5 and 10 days) of the ASVs were determined with the betapart package (v1.5.6) [40].

Ordination (principal component analysis (PCA)) and multivariate comparison (perMANOVA) were done with converted sequence data using the centered log-ratio transformation test, i.e. clr-transformation, returned by the aldex.clr argument with the ALDEx2 package (v1.21.1) [41]. Centered log-ratio transformation of all sequence data is necessary as all high-throughput data are compositional. The effect of fumigation and time (0, 1, 5 and 10 days) on bacterial groups was visualized with a PCA as given by the FactoMineR package (v2.3) [42]. The significance of fumigation and time on the ASVs was determined with a permutational multivariate analysis of variance (perMANOVA) test with the vegan package (v2.5–7) [43]. Permutation multivariate analysis of dispersion (PERMDISP) was computed using the betadisper function from the vegan package (v2.5–7 [43]), to determine dispersion within soils when the perMANOVA test indicated a significant effect of fumigation or time on bacterial groups. The expected p-value of the Kruskal-Wallis test calculated with the Package ALDEx2 (v1.21.1) [41] was used to determine the effect of fumigation on bacterial groups. Effect size, i.e. the difference between relative abundance of the bacterial groups divided by maximum dispersion within the treatment, was determined with the Package ALDEx2 (v1.21.1) [41]. The effect size was plotted versus the expected p-value of the Kruskal-Wallis test for each feature in a volcano plot.

### Accession Number of the 16 S rRNA Gene Sequence Datasets

The 16 S rRNA gene sequence datasets were submitted to the NCBI Sequence Read Archive (SRA) under BioProject accession number PRJNA1313659 (https://www.ncbi.nlm.nih.gov/sra/PRJNA1313659).

## Results

### Soil Characteristics

The sandy clay loam soils with an average pH 10.2 and EC 146 dS/m, had an organic C content that ranged from 22.6 to 46.7 g/kg and total N content from 0.9 to 1.4 g/kg resulting in a high C: N ratio between 25 and 34 (Table 1). The microbial biomass C ranged from 67 to 106 mg C/kg, which was < 0.02% of the soil organic C.

**Table 1:** Some selected characteristics of soil sampled at the former lake Texcoco (Mexico) as given in Bello-López et al. (2014)

		EC ^a^	Organic C	Total N	Microbial C		Ratio microbial-C	WHC ^b^	Clay	Loam	Sand	USDA soil Classification
Sample	pH	(dS m^-1^)	—(g C kg^-1^)—		C:N ratio	(mg C kg^-1^)	organic-C (´ 10^-3^)	—(g kg^-1^)—				
Soil 1	10.2	157	46.7	1.4	33	67	1.4	747	480	130	390	Clay
Soil 2	10.0	143	30.9	0.9	34	81	1.7	923	330	90	580	580 Sandy Caly loam
Soil 3	10.3	139	22.6	0.9	25	106	2.3	850	330	210	460	460 Sandy Caly loam

^a^EC: Electrolytic conductivity, ^b^ WHC: Water holding capacity.

### Alpha and Beta Diversity of the Archaeal Community

**Fig. 1:**
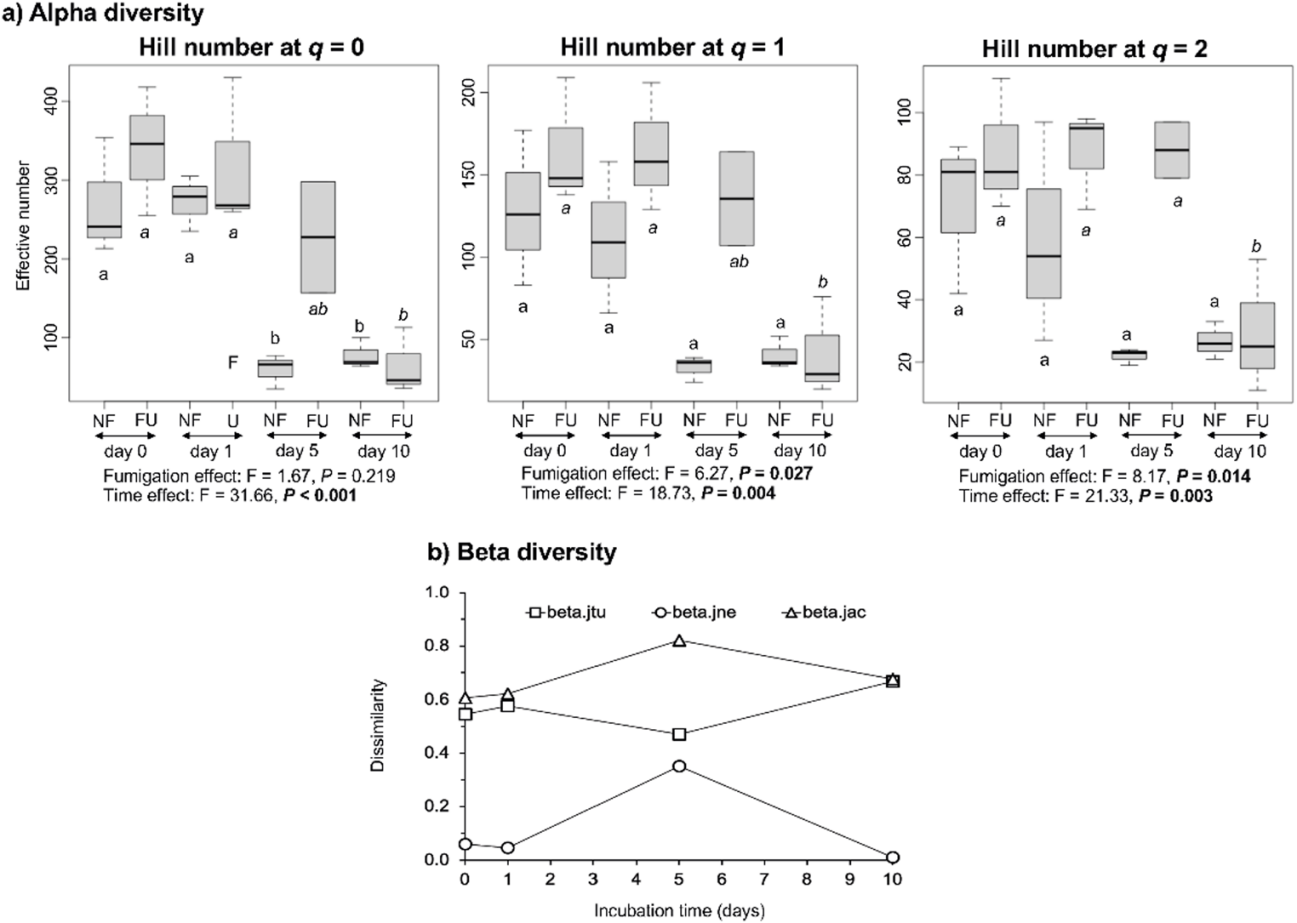
**a**) Hill numbers at *q* = 0, 1 and 2 in the fumigated and unfumigated soil incubated aerobically for 10 days with values with a different letter indicating a significant difference in the unfumigated soil over time and with a different letter in italic indicating a significant difference in the fumigated soil over time and **b**) Beta-diversity of the effect of fumigation and changes over time of the amplicon sequence variants (ASVs) with beta.jtu: dist dissimilarity matrix accounting for spatial turnover, measured as the turnover-fraction of Jaccard pair-wise dissimilarity, i.e. indicates 1-for-1 species substitutions, beta.jne: dist object, dissimilarity matrix accounting for nestedness-resultant dissimilarity, measured as the nestedness-fraction of Jaccard pair-wise dissimilarity, i.e. indicates species gain or loss without substitution, and beta.jac: dist object, dissimilarity matrix accounting for beta diversity, measured as Jaccard pair-wise dissimilarity (a monotonic transformation of beta diversity), i.e. the full jaccard index with values closer to 1 indicate greater dissimilarity [40]

The Hill numbers *q* = 0, 1 and 2 were significantly affected by time and those at *q* = 1 and 2 by fumigation (Fig. 1a). The Hill numbers at *q* = 0 were lower in the unfumigated soil on 5 and day 10, and on day 10 in the fumigated soil than on day 0 and 1. The Hill numbers at *q* = 1 and *q* = 2 were lower in the fumigated soil on day 10 than on day 0 and 1.

The beta diversity analysis indicated that most changes in the archaeal community due to fumigation were the result of 1-to-1 replacement (turnover) (Fig. 1b). At day 5, some loss of ASVs (nestedness) also occurred and the dissimilarity was higher.

### The Archaeal Community Structure in the Extreme Alkaline Saline Soil

**Fig. 2:**
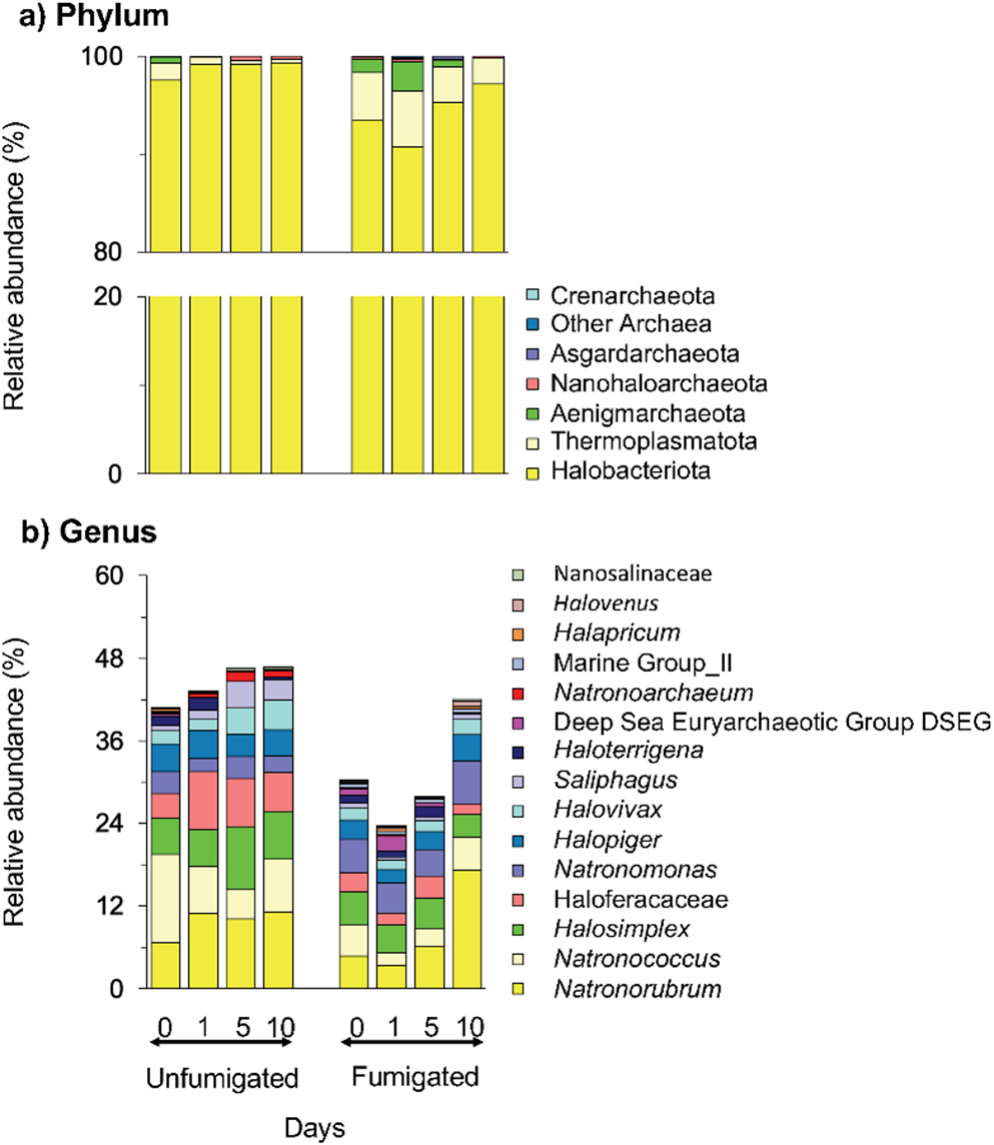
Relative abundance of the different archaeal **a**) phyla and **b**) genera in the fumigated and unfumigated soil incubated aerobically for 10 days

Six different archaeal phyla were detected in the unfumigated soil, with members of Halobacterota being the most abundant (96.5%) (Fig. 2a). *Natronorubrum* was the most dominant genus in both unfumigated (9.7%) and fumigated (7.9%) soils. In the unfumigated soil, the second most abundant genus was *Natronococcus* (7.9%), whereas in the fumigated soil it was *Natronomonas* (4.8%) (Fig. 2b). The most abundant ASV in the unfumigated soil belonged to the phylum Candidatus Halobacterota (6.4%) and an uncultured *Natronorubrum* (2.6%) in the fumigated soil.

**Fig. 3:**
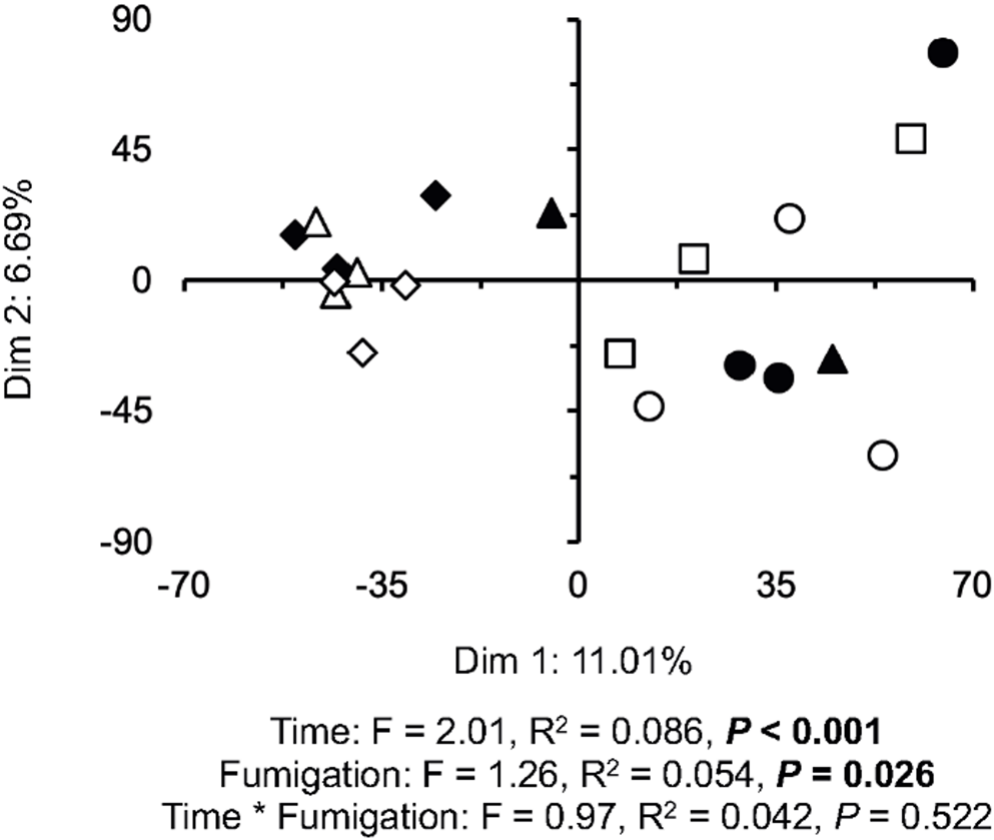
Principal component analysis considering the different archaeal groups found in unfumigated soil at the onset of the experiment (◼), after 1 day (⬤), 5 (▲) and 10 days (◆), and in the unfumigated soil at the onset of the experiment (☐) and after 1 day (○), 5 (△) and 10 days (◇)

**Fig. 4:**
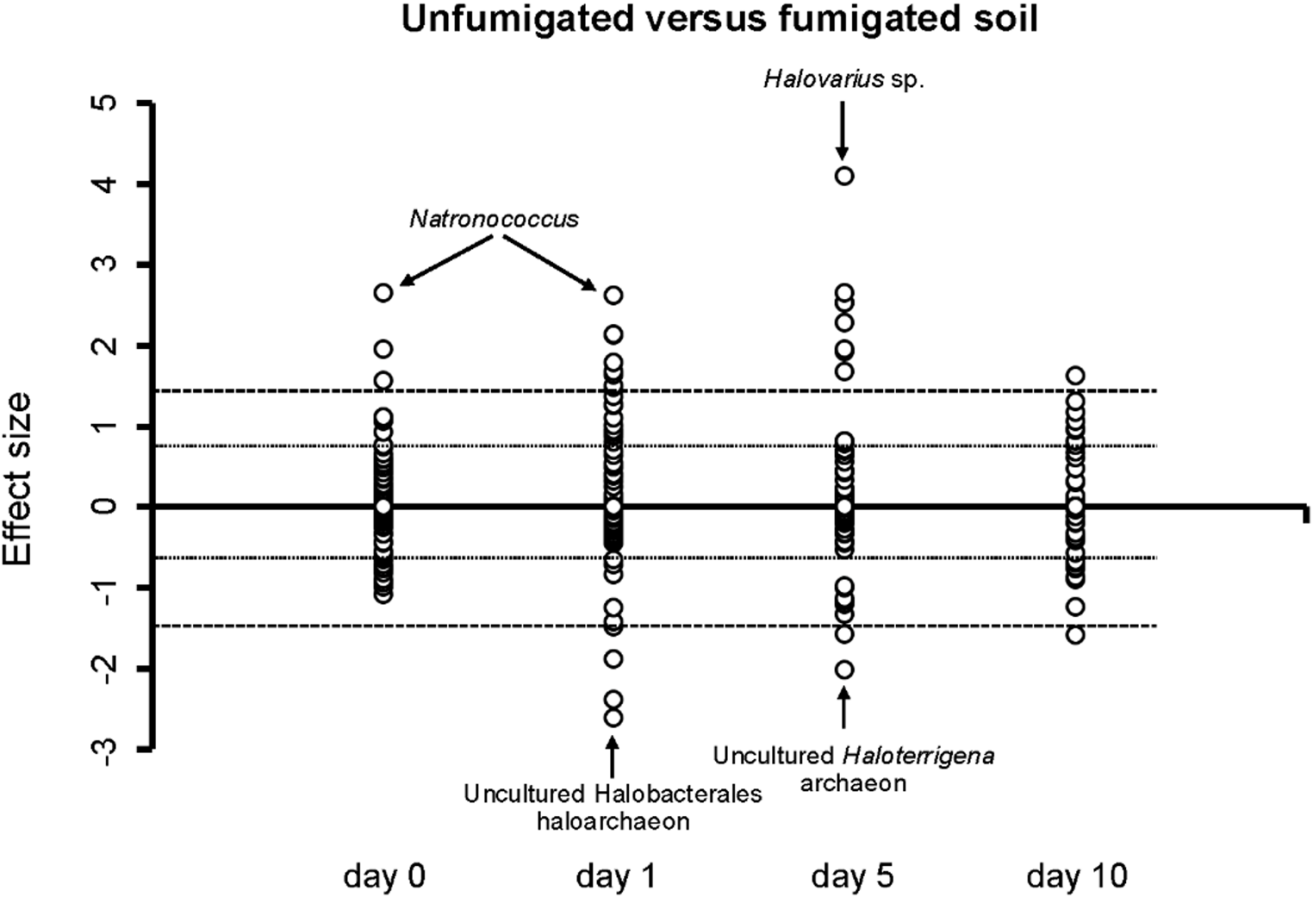
Effect size of all groups assigned up to the taxonomic level of genus in the unfumigated versus the fumigated soil incubated aerobically at 22 ± 2 °C for 10 days. The effect size, which is defined as the difference between groups divided by the maximum dispersion within group A or B, was calculated with the ALDEx2 package using the aldex.ttest argument. A positive value indicates that the relative abundance of the microbial group was higher in the unfumigated compared to the fumigated soil while a negative value indicates the opposite. Vertical lines indicate large (≤ −0.8, ≥ 0.8) and very large effect sizes (≤ −1.3, ≥ 1.3)

The PCA did not separate clearly the unfumigated from the fumigated soil although the effect was significant (*P* = 0.026) and the dispersion was not (*P* = 0.858) (Fig. 3). There was a clear effect of time on the archaeal community structure considering the ASVs and it was highly significant (*P* < 0.001). The samples of day 5 and 10 of the unfumigated soil were grouped with the fumigated samples after 10 days and most of those on day 5 in the upper and lower left quadrants, while those of day 0 and 1 were found in the upper and lower right quadrants. Fumigation had a large effect on some archaeal groups most accentuated on day 1 and 5, and less so on day 10 (Figs. 4, S2, Table 2).

**Table 2:** Effect of fumigation on the relative abundance of archaeal genera and amplicon sequence variants (ASVs)

Comparing the relative abundance of the archaeal groups in the unfumigated versus the fumigated soil. A positive effect size means that the relative abundance was higher in the unfumigated than in the fumigated soil, while a negative value the opposite.
**Day 0** Uncultured* Natronococcus* archaeon (1.6 ^a, b^), uncultured *Halopiger* haloarchaeon (2.0), *Natronococcus* (2.7) **Day 1** Uncultured Halobacterales haloarchaeon (-2.6), Uncultured Thermoplasmata archaeon (-2.4), Uncultured Halomicrobiaceae archaeon (-1.9), Deep Sea Euryarchaeotic Group (DSEG) (-1.5), uncultured Marine Group II archaeon (-1.4), Uncultured *Natronorubrum* archaeon (1.4), Uncultured *Natronorubrum* haloarchaeon (1.5), Uncultured *Haloterrigena* archaeon (1.5), *Natronorubrum* (1.6), Uncultured *Halosimplex* archaeon (1.7), uncultured Halobacteriales archaeon (1.8), *Halovarius* sp. (2.1), uncultured *Halopiger* haloarchaeon (2.1), *Natronococcus* (2.6)
**Day 5** Uncultured *Haloterrigena* archaeon (-2.0), Uncultured Halomicrobiaceae archaeon (-1.6), Uncultured *Halovivax* archaeon (1.7), *Natronorubrum* (1.9), *Natronococcus* (2.0), Uncultured *Natronorubrum* archaeon (2.3), Uncultured *Natronoarchaeum* archaeon (2.5), Uncultured *Halopiger* haloarchaeon (2.5), Uncultured *Halosimplex* archaeon (2.7), *Halovarius* sp. (4.1) **Day 10** Uncultured Halobacterales haloarchaeon (-1.6), Uncultured Haloferacaceae haloarchaeon (1.6)

^a^ Only archaeal groups with an effect size ≥ 1.4 or ≤ -1.4 are given, ^b^ The effect size, which is defined as the difference between groups divided by the maximum dispersion within group A or B, was calculated with the aldex.ttest argument (ALDEx2 (version, 1.18), Gloor et al. (2020).

## Discussion

### The Archaea in an Extreme Alkaline Saline Soil

Archaea play an important role in soil as they participate in the carbon cycle [44] (Yang et al., 2022). Archaea can fix carbon and produce methane in anaerobic conditions but also anaerobically oxidize it. Archaea also play an important role in the nitrogen cycle as oxidizers of ammonium [45] (Huang et al., 2021) and in the sulfur cycle [46] (Wasmund et al., 2017). Six different archaeal phyla were detected in the Texcoco soil with Candidatus Halobacterota the most dominant (taxonomic classification based on Rinke et al. [47] (96.9%, mostly Halobacteria class 96.5%). Halobacteria are exceptionally well adapted to extreme halophilic conditions containing different genes encoding for stress proteins [48] which might explain their high relative abundance in the extreme alkaline saline soil of the former lake Texcoco. The second most abundant phyla, i.e. Thermoplasmatota (2.2%), has been found in different ecosystems. Some clades belonging to this phylum have the capacity to recycle complex organic C and some of them, such as Methanomassiliicoccales, are methanogens [49]. All Thermoplasmatota detected in the soil of Texcoco were uncultured, including archaea or haloarchaea members of the Marine-Group-II also called planktonic Poseidoniales [50]. They are mostly found in marine environments that metabolize dissolved organic matter, such as lipids and peptides and are photoheterotrophs [51]. They were described by DeLong [52] as “*unexpected ubiquitous*,* with unusual symbiotic associations*,* unpredicted physiologies and biogeochemistry*,* and global abundance*”. Our results suggest that their distribution might even be wider and that they might also be found in saline alkaline lakes or their sediments.

The Aenigmarchaeota the third most abundant archaeal phylum (0.63%) belong to the DPANN superphylum. This is an acronym of the names of the first included phyla Diapherotrites, Parvarchaeota, Aenigmarchaeota, Nanohaloarchaeota and Nanoarchaeota [53]. The Aenigmarchaeota are symbionts that might rely on a wide range of hosts [54]. Extremely halophilic members of *Candidatus* Nanohaloarchaeota (0.21%, DPANN superphyla) are obligately associated with extremely halophilic Archaea of the phylum *Halobacteriota* [55]. Their adaptation to high salinity and their association with Halobacteriota the most abundant archaeal phyla explained why they were detected in extreme alkaline saline soil of the former lake Texcoco. In this study, all members of the phylum *Candidatus* Nanohaloarchaeota (0.20%) belonged to Nanosalinaceae with *Candidatus* Haloredivivus, first detected in a hypersaline saltern pond, the only assigned genus [56].

Members of the phylum Asgardarchaeota share numerous eukaryotic-like characteristics [57]. An uncultured archaeon of the genus *Heimdallarchaeia* was the only species of the Heimdallarchaeota detected in Texcoco soil. Heimdallarchaeota is one of the dominant Asgardarchaeota clades found in saline habitats where they might employ anaerobic/microaerophilic organic matter degradation and autotrophic carbon fixation [58]. In contrast, Crenarchaeota are often dominant in agricultural, rhizosphere and forest soils [59], but inhibited in extreme environments [60]. This might explain why they were absent from most fumigated and unfumigated samples (0.005%) in the Texcoco soil. The ASVs of the Crenarchaeota detected in the soil of Texcoco belonged to the Nitrosopumilaceae with some of its members described as autotrophic ammonia-oxidizing marine Archaea [61] and the Nitrososphaeraceae ammonia oxidizing Archaea isolated from soil [62]. They might explain together with bacterial nitrifiers why oxidation of NH_4_^+^ occurred in these alkaline saline soils.

Members of *Halosimplex*,* Natronococcus* and *Natronorubrum* were the most abundant archaeal genera in the soil of Texcoco. They are well known halophilic Archaea. Most species that belong to *Halosimplex* (e.g. *H. halophilum and H. salinum*), *Natronococcus (N. pandeyae*) and *Natronorubrum* (*N. texcoconense*) have been isolated from saline or alkaline saline environments such as soil of the former lake Texcoco [63].

### Fumigation and its Effect on Archaea

How Archaea and Bacteria respond to a disturbance depends on the type, the intensity and its duration [5]. Lv et al. [64] reported that the “*abundance and diversity of bacterial communities in river sediments were more sensitive to anthropogenic and naturally induced environmental changes than that of archaeal communities*”. On the one hand, bacterial communities showed greater resistance to long-term disturbances than archaeal communities, such as seasonal changes, because of a more complex community structure and a larger species diversity. Archaea, however, were more resistant to short-term drastic environmental disturbances, such as water transfer, as they were less sensitivity to environmental changes than Bacteria. However, the type of disturbance will also determine how bacteria or archaea are affected by the event. For instance, Jurburg et al. [65] found that AOA were sensitive to cold shocks, whereas AOB were not; the latter were sensitive to heat shock.

Fumigation had a strong effect on the bacterial community structure and their relative abundance [18]. The Bacteria that recolonized the soil were different from those in the inoculum [18]. The archaeal community structure, however, was not affected in the same way by fumigation, i.e. there was a small effect of fumigation on the archaeal community structure but a large effect of time of incubation. This might be related to a general characteristic of Bacteria and Archaea. The Bacteria dominating in the fumigated soil were mostly well-known spore-formers and metabolic versatile, i.e. Bacillota (formerly Firmicutes). They became active after fumigation and dominated the bacterial population [18]. The factors determined which Archaea recolonized the soil might have been different from those important for the recolonizing Bacteria. A first possible explanation is that most of the Archaea were killed by chloroform fumigation, so the recolonizing Archaea came solely from the added inoculum, i.e. they were similar as in the unfumigated soil. Second, most of the Archaea might have survived the chloroform fumigation. Most characterized Archaea and many Bacteria have a proteinaceous cell wall, i.e. the S-layer [66]. It consists of a regularly structured two-dimensional array based on a single protein species, the S-layer glycoprotein, or a limited number of proteins [67]. The S-layer glycoproteins and other glycoproteins, such as archaellins and pilins, of Archaea have a wide range of N-linked glycans and this N-glycosylation has been thought to aid Archaea in coping with extreme environments they often inhabit [67]. Third, the larger stability of some archaeal proteins and enzymes might also explain why chloroform had a smaller effect on the archaeal community structure than it had on the bacterial community structure, i.e. their enzymes and proteins are stable under extreme conditions while those of other microorganisms.

Aenigmarchaeota were absent in the unfumigated soil after 5 and 10 days, but reached 2.99% in the fumigated soil at day 1 and were not detected after 10 days. This would suggest that their hosts were apt at recolonizing the soil and involved in the degradation of the lysed microbial cells [54].

Fumigation had a limited effect on members of *Halosimplex*, reduced the relative abundance of *Natronococcus* and enriched that of *Natrorubrum* after 10 days. This would suggest that members of *Natronococcus* have a more oligotrophic lifestyle, i.e. they are enriched in nutrient poor environments. However, most of the Archaea enriched after fumigation, i.e. those that recolonized the fumigated soil with a copiotrophic lifestyle, were uncultured.

### Changes Over Time

The archaeal community structure changed over time and became similar in the fumigated and unfumigated soil after 10 days. It is difficult to speculate what might have caused these changes, but changes in the availability of organic material might be responsible. Mixing a soil releases organic material as soil aggregates are broken up and physically protected organic material becomes available as C substrate altering the bacterial community structure. This rapid change in the concentration of easily decomposable soil organic material might have affected the archaeal community. Additionally, mixing the soil might have broken up soil aggregates changing micro-environmental conditions exposing the Archaea to changed conditions. After 10 days, this effect on the archaeal community structure became negligible.

## Conclusion

Fumigation had no significant effect on the archaeal richness (Hill number at *q* = 0), but it changed significantly over time in the unfumigated and fumigated soil. Six different archaeal phyla were detected in the extreme saline alkaline soil with an average pH 10.2 and EC 146 dS/m dominated by Halobacterota (mostly Halobacteriaceae). *Natronorubrum* dominated in the unfumigated (9.7%) and fumigated soil (8.9%), while *Natronococcus* (7.9%) was the second most abundant genus in the unfumigated soil and *Natronomonas* (4.8%) in the fumigated soil. Interestingly, ASVs of 14 uncultured Archaea of the Marine-Group-II or planktonic Poseidoniales, normally found in the ocean, were detected in the extreme saline soil of the former lakebed. Chloroform fumigation had a significant but short-term effect on the archaeal community structure and, after 10 days, the archaeal community structure in the fumigated soil resembled already that in the unfumigated soil. The specific structure of the archaeal membrane and cell wall might have limited the effect of the chloroform fumigation on the archaeal community structure. Time had a highly significant effect on the archaeal community structure. Some archaeal groups were strongly affected by fumigation most accentuated on day 1 and 5. The short-term increases in the relative abundance of some archaeal groups might have been related to their capacity to metabolize killed soil microorganisms. It would be interesting to investigate if some archaeal groups replaced bacteria as archaea were more resistant to the disturbance than bacteria. It is important to remember that this experiment was based on PCR amplification of archaeal 16 S rRNA genes. Other studies in other extreme environments based on other techniques, e.g. shotgun metagenomics, will be necessary to confirm the results obtained in this study.

## Supplementary Information

Below is the link to the electronic supplementary material.

Supplementary Material 1

Supplementary Material 2

## Data Availability

The 16 S rRNA gene sequence datasets were submitted to the NCBI Sequence Read Archive (SRA) under accession number SUB796334. Other data will be made available on request.
