# Supplementary figures and images for: Resistance of the Archaeal Community to a Severe Disturbance in an Extreme Alkaline Saline Soil
Source: Curr Microbiol. 2026 May 13;83(7):358. doi: 10.1007/s00284-026-04895-1 (PMC13171702; doi:10.1007/s00284-026-04895-1)

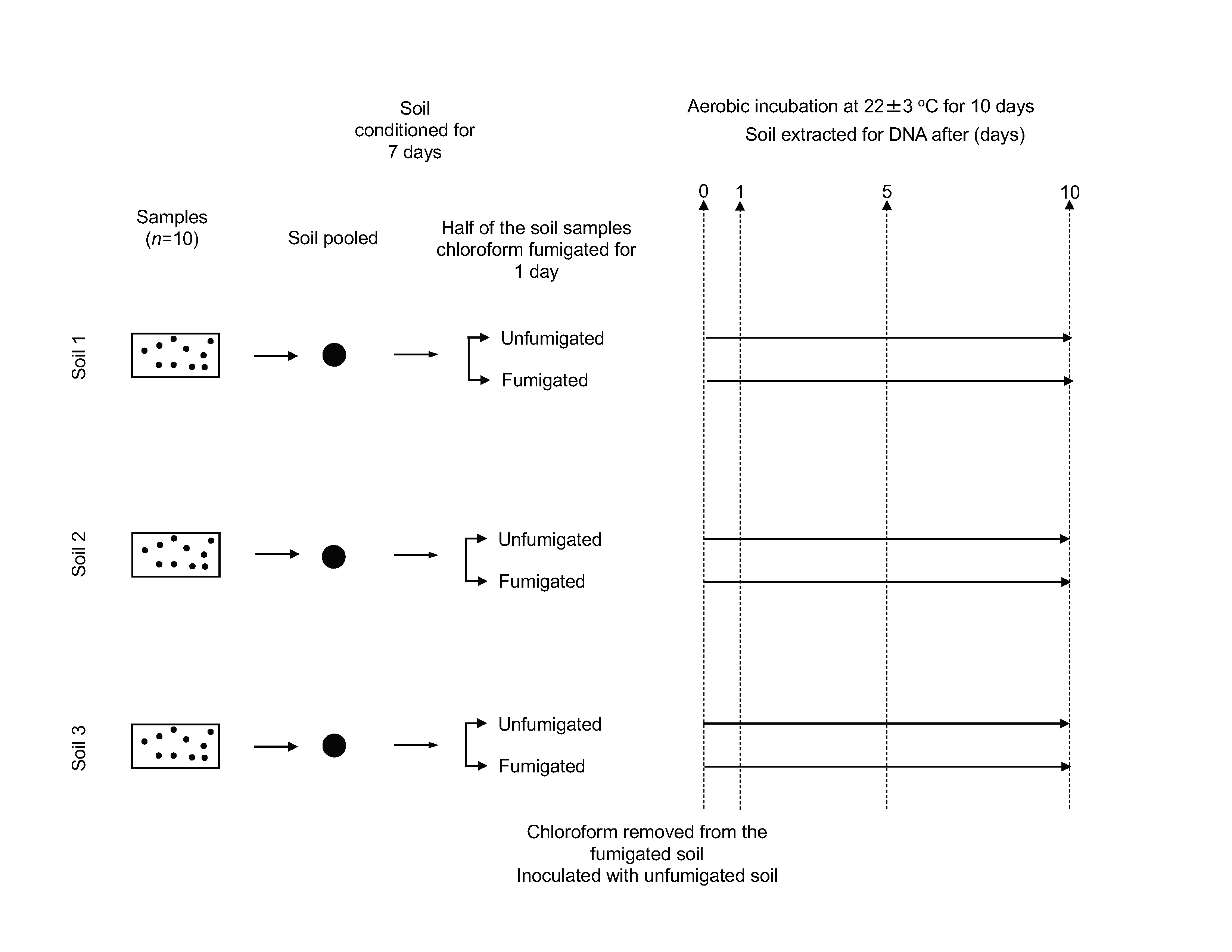

Supplement: Supplementary file 1 — Supplementary Material 1 [file 284_2026_4895_MOESM1_ESM.tiff]

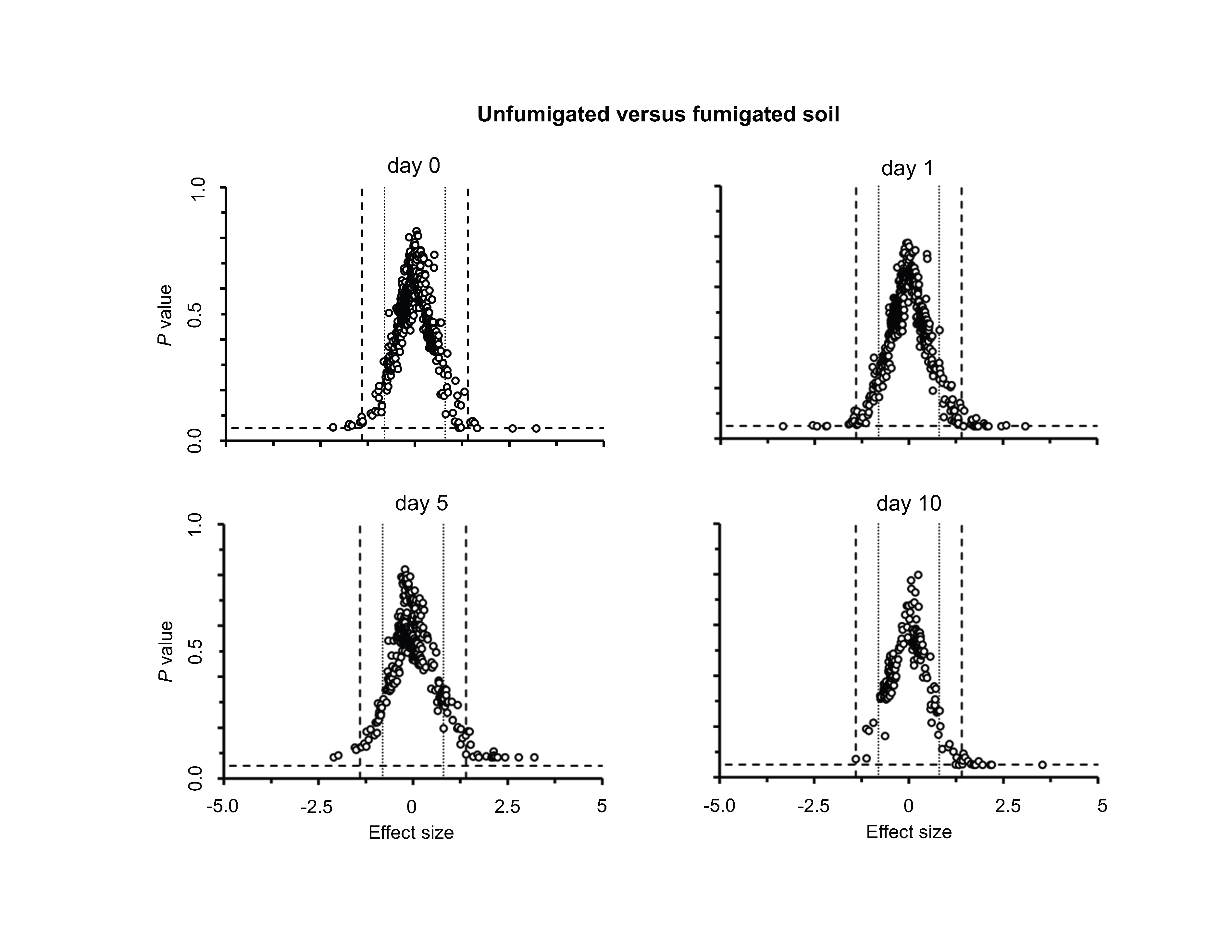

Supplement: Supplementary file 2 — Supplementary Material 2 [file 284_2026_4895_MOESM2_ESM.tiff]
